# Toward Marrow Adipocytes: Adipogenic Trajectory of the Bone Marrow Stromal Cell Lineage

**DOI:** 10.3389/fendo.2022.882297

**Published:** 2022-04-22

**Authors:** Yuki Matsushita, Wanida Ono, Noriaki Ono

**Affiliations:** University of Texas Health Science Center at Houston School of Dentistry, Houston, TX, United States

**Keywords:** single-cell RNA sequencing (scRNAseq), skeletal stem cells (SSCs), *in vivo* lineage-tracing experiments, bone marrow stromal cell (BMSC), bone marrow adipose tissue (BMAT)

## Abstract

Bone marrow contains precursor cells for osteoblasts and adipocytes in the stromal compartment. Bone marrow adipose tissue (BMAT) is an important constituent of the bone marrow that is particularly abundant in adults. BMAT is composed of the proximal “regulated” BMAT containing individual adipocytes interspersed within actively hematopoietic marrow, and the distal “constitutive” BMAT containing large adipocytes in the area of low hematopoiesis. Historically, bone marrow adipocytes were regarded as one of the terminal states of skeletal stem cells, which stand at the pinnacle of the lineage and possess trilineage differentiation potential into osteoblasts, chondrocytes and adipocytes. Recent single-cell RNA-sequencing studies uncover a discrete group of preadipocyte-like cells among bone marrow stromal cells (BMSCs), and recent mouse genetic lineage-tracing studies reveal that these adipocyte precursor cells possess diverse functions in homeostasis and regeneration. These adipogenic subsets of BMSCs are abundant in the central marrow space and can directly convert not only into lipid-laden adipocytes but also into skeletal stem cell-like cells and osteoblasts under regenerative conditions. It remains determined whether there are distinct adipocyte precursor cell types contributing to two types of BMATs. In this short review, we discuss the functions of the recently identified subsets of BMSCs and their trajectory toward marrow adipocytes, which is influenced by multiple modes of cell-autonomous and non-cell autonomous regulations.

## Introduction

Bone marrow houses diverse classes of cells, including cells in the skeletal (or mesenchymal), the hematopoietic (blood) and the endothelial (vascular) lineages. Bone marrow contains precursor cells for bone-making osteoblasts and lipid-accumulating adipocytes in the stromal compartment, both of which are considered to play important roles in bone homeostasis and regeneration, as well as in hematopoiesis by providing a microenvironment. Classically, skeletal stem cells with self-renewability and multipotency are considered to stand at the top of the lineage, and their descendants fall through a hierarchical model, differentiating first into progenitors then into terminally differentiated cells such as osteoblasts or adipocytes (1). However, it remains largely undefined whether skeletal stem/progenitor cells can be defined as discrete cell populations within the continuous spectrum of the bone marrow stromal cell lineage, and how their relationships with osteoblasts and adipocytes develop in a complex bone marrow microenvironment.

Recent single-cell RNA-sequencing (scRNA-seq) studies reveal profound cellular heterogeneity within bone marrow stromal cells (BMSCs) that are isolated by fluorescence-activated cell sorting (FACS) (2–9). These studies consistently found discrete preadipocyte-like cells populations expressing *leptin receptor* (*Lepr*) and *C-X-C motif chemokine ligand 12* (*Cxcl12*) within BMSCs. These cells highly express classical adipocyte markers such as *adiponectin* (*Adipoq*) and adipogenic transcription factors such as *peroxisome proliferator activated receptor gamma* (*Pparg*). These adipogenic cell populations encompass newly defined subsets of Adipo-CXCL12 abundant reticular cells (Adipo-CAR cells) (10, 11) and marrow adipogenic lineage precursors (MALPs) (12), which mainly exist as non-proliferative pericytes and perivascular stromal cells. Recent mouse genetic studies highlight the diverse functions of bone marrow adipocyte precursor cells that not only contribute to the formation of bone marrow adipose tissues (BMATs) but also regulate the formation of the trabecular and cortical bones (for more detailed reviews, see (13, 14)).

In this short review, we discuss the functions of bone marrow adipocyte precursor cells in homeostasis and regeneration, and potentially diverse cellular sources of bone marrow adipocytes.

## Bone Marrow Adipose Tissue (BMAT) and Its Function

Functionally, bone marrow adipocyte precursor cells are poised to differentiate into lipid-laden adipocytes that generate bone marrow adipose tissues (BMATs). Marrow adiposity increases under various physiological and pathological conditions such as aging, osteoporosis, radiation, chemotherapy (15). BMAT represents a unique form of adipose tissues that constitutes over 10% of the total fat mass in lean and healthy human adults (16). BMAT is composed of regulated and constitutive BMATs (rBMAT and cBMAT, respectively) with distinct functionality (17). rBMAT is mainly located in the proximal skeletal components and contains individual adipocytes interspersed within the areas of active hematopoiesis. In contrast, cBMAT is mainly located in the distal portion and contains large adipocytes that develop in the areas of low hematopoiesis; the latter cBMAT develops earlier and remains preserved upon systemic challenges (17–19).

Historically, the two types of bone marrow, “red marrow” and “yellow marrow”, have been recognized for several decades (20). The “red marrow” consists of blood-forming cells with scattered adipocytes, whereas the “yellow marrow” is filled almost entirely with adipocytes (21, 22). The cBMAT starts to form in the distal area at prenatal to neonatal stages, followed by rapid expansion early in life (17). In contrast, rBMAT develops later and expands with age, generally in areas of active hematopoiesis (17). BMAT acts as an endocrine organ and energy storage depot that can contribute to bone homeostasis, metabolism, hematopoiesis, and cancers (23), and associates with the pathophysiology of bone diseases such as osteoporosis (24). For example, osteoporosis is commonly associated with increased BMAT (25), and adipocyte-derived factors from BMAT can suppress osteoblast differentiation of skeletal stem cells and regulate bone remodeling (26). Interestingly, lipid metabolism of BMAT is distinct from that of subcutaneous adipocytes, as bone marrow adipocytes show diminished lipolytic activities and exhibit cholesterol-directed metabolism (27, 28). BMAT also has distinct roles in glucose homeostasis (29, 30). Moreover, bone marrow adipocytes regulate hematopoiesis through direct contact and cytokine secretion. In fact, marrow adipogenesis is associated with impaired hematopoiesis (31). Bone marrow adipocytes also regulate the progression of hematological diseases (32) and hematopoietic microenvironment (HME) regeneration (33), and promote proliferation and bone metastasis of cancer cells including prostate and breast cancers and melanoma (34–37). Therefore, BMAT has important regulatory functions in bone metabolism, hematopoiesis and bone metastasis.

Despite unique metabolic status and functions, whether rBMAT and cBMAT are supported by distinct populations of precursor cells remains unknown. Subcutaneous and marrow adipocytes are derived from different precursor cell populations and possess different metabolic patterns. It is important to characterize marrow adipocyte precursor cells further to unravel molecular mechanisms supporting the unique functions of distinct classes of BMATs.

## The Bone Marrow Stromal Cell Lineage and Its Trajectory Toward Marrow Adipocytes

Understanding the landscape of the bone marrow stromal cell lineage is essential to identifying potential cellular origins of BMATs. Cells constituting the bone marrow stromal cell lineage have been at least partly revealed by recent large-scale scRNA-seq studies of BMSCs that are isolated by cell sorting (2–5, 11). The major limitation of these single-cell approaches is that bone marrow adipocytes are large-sized (~150µm) and fragile, therefore cannot be captured through conventional cell sorting or encapsulated in oil droplets in microfluidic devices. Nonetheless, these scRNA-seq studies successfully have identified discrete clusters of adipocyte precursor cells (preadipocyte-like cells) that abundantly express *Lepr*, in addition to cell clusters that might correspond to skeletal stem and progenitor cells, preosteoblasts and other stromal cell types.

These studies further infer the potential lineage relationship among identified bone marrow stromal cell types using computational approaches, such as RNA velocity (3, 4, 11). These studies identify putative skeletal stem cell populations, such as *Lepr^+^* (encoding LepR) (3), *Cspg4^+^* (encoding NG2) (11) or *Nt5e^+^* (encoding CD73) (4), which are predicted to provide a cellular source of adipocyte precursor cells. Importantly, these studies place osteoblasts and preadipocytes at the opposite ends of the inferred lineage trajectory, while simultaneously identifying a number of transitional cell types among putative terminal states, highlighting the contiguous nature of the bone marrow stromal cell lineage that spans over osteoblasts and marrow adipocytes.

Therefore, recent scRNA-seq studies corroborate with the well-established ideas that preadipocyte-like cells represent a distinct state from osteoblasts and their precursors among BMSCs. However, the identities of putative skeletal stem/progenitor cell populations are variable among studies, therefore remain largely ambiguous. Further studies are required to delineate the identities of these stem cells and their relationships with more abundant preadipocyte-like stromal cells.

## Adipogenic Subset of CXCL12^+^LepR^+^ Cells (Adipo-CAR Cells) and Their Osteogenic Functions

As mentioned above, cells abundantly expressing adipocyte-related markers such as *Lepr* and *Adipoq* constitute major cellular subsets of BMSCs (5, 11). *Lepr* encodes leptin receptor (LepR) that is a cognate receptor for the circulating adipokine, leptin. LepR^+^ stromal cells overlap substantially with CXCL12-abundant reticular (CAR) cells, as cells marked by Cxcl12-GFP coincide (approximately 90%) with cells marked by *Lepr-cre* (10, 38). LepR^+^ stromal cells provide a major source of adipocytes in adult bone marrow (38), which is further supported by more recent lineage-tracing studies using *Lepr-creER* (39). Functionally, LepR promotes marrow adipogenesis, as conditional deletion of LepR in BMSCs using *Prrx1-cre* increases osteogenesis and decreases adipogenesis in bone marrow (40).

CAR cells have been originally described as adipo-osteogenic progenitors that form a component of the hematopoietic stem cell niche (41). CAR cells can be uniformly marked by *Ebf3-creER* in the adult stage (Ebf3^+^ CAR cells), and contribute to both osteoblasts and adipocytes (42), supporting that LepR^+^ and CXCL12^+^ cells have similar cell fates. Importantly, CXCL12 is most abundantly expressed by reticular stromal cells in the central marrow space, and plays important roles in maintaining hematopoietic stem and progenitor cells (43–49). In fact, CXCL12 deletion in BMSCs causes reduction in hematopoietic cells in bone marrow (50, 51).

**Figure 1:**
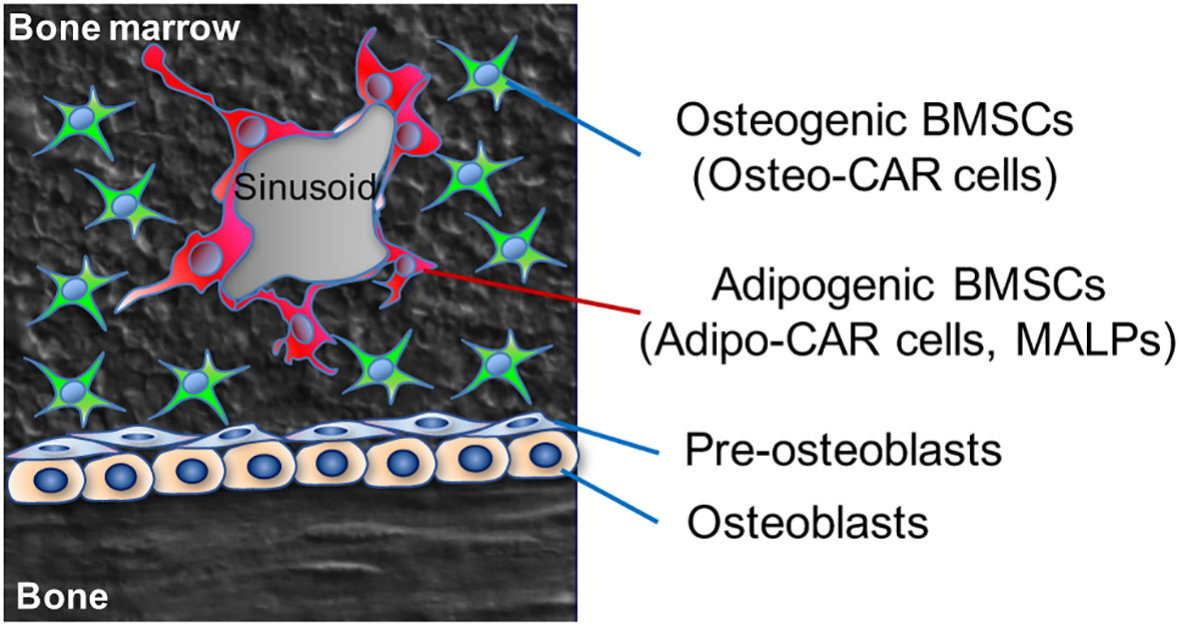
Two major subsets of bone marrow stromal cells (BMSCs). Adipogenic BMSCs (Adipo-CAR cells) with pre-adipocyte-like properties are localized to the areas around sinusoidal vessels located in the center of the marrow space, which are poised to differentiate into adipocytes.

CAR cells have been recently reclassified into two classes of Osteo-CAR and Adipo-CAR cells (11), instead of a uniform entity as adipo-osteogenic progenitors (**Figure 1**). Osteo-CAR cells express both *Cxcl12* and *Alpl* (encoding alkaline phosphatase, a preosteoblast marker), but not *Lepr*. These Osteo-CAR cells are localized to arterioles and in proximity to the bone surface, which are poised to differentiate into osteoblasts. In contrast, Adipo-CAR cells with pre-adipocyte-like properties are localized to the areas around sinusoidal vessels located in the center of the marrow space, which are poised to differentiate into adipocytes. Adipo-CAR cells abundantly express classical adipocyte markers (*Adipoq*, *Lepr*) as well as hematopoiesis-supporting cytokines (*Kitl* and *Cxcl12*), but do not express osteogenic markers and cytokines such as *Alpl*, *Sp7* and *Clec11a* at the same level. It is intriguing to speculate that the adipocyte precursor identity may confer these Adipo-CAR cell with some metabolic advantages to secrete large amounts of cytokines, such as Kit ligand (SCF), CXCL12 and Adiponectin.

The next important question is, what are the *in vivo* cell fates and functions of preadipocyte-like BMSCs including Adipo-CAR cells? The answer to this question has been partly contributed by mouse genetic lineage-tracing studies using a *Cxcl12-creER* line (10). Interestingly, the *Cxcl12-creER* bacterial artificial chromosome (BAC) transgenic line can almost exclusively mark a preadipocyte-like subset of CAR cells, which may correspond to Adipo-CAR cells, upon tamoxifen injection. These cells are quiescent and dormant with little colony-forming activities in physiological conditions. These Cxcl12-creER^+^ preadipocyte-like cells readily become marrow adipocytes, but do not become cortical bone osteoblasts in normal conditions. However, these Cxcl12-creER^+^ cells can rapidly convert their identity into a skeletal stem cell-like state in response to injury, associated with upregulation of osteoblast-signature genes and activation of canonical Wnt signaling components. As a result, these cells further differentiate into cortical bone osteoblasts to repair bone defects.

**Figure 2:**
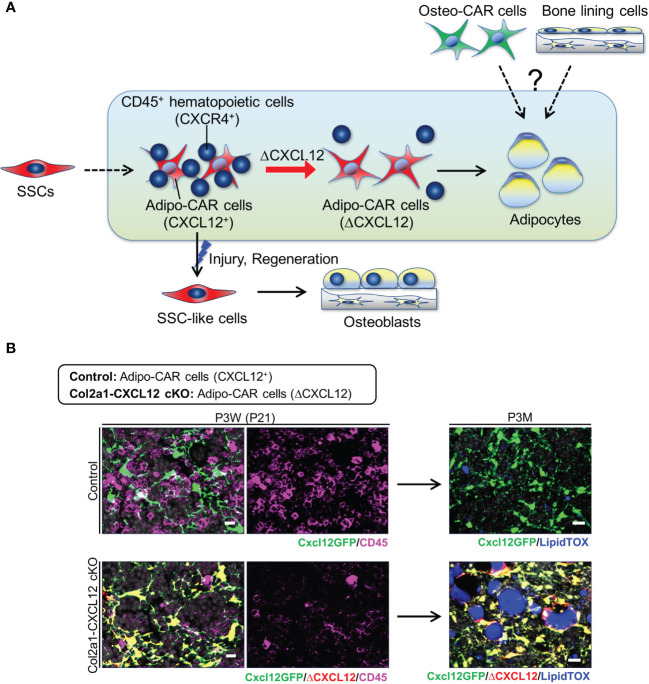
Adipogenic trajectory of the bone marrow stromal cell lineage. **(A)** In physiological condition, dormant Adipo-CAR cells can become marrow adipocytes. Hematopoietic cells may regulate marrow adipogenesis in a manner dependent on CXCL 12-mediated physical coupling. CXCL 12 deletion leads to a reduction of stromal-hematopoietic coupling and accelerates marrow adipogenesis. In contrast, Adipo-CAR cells convert into skeletal stem cell-like cells in response to injury, and redifferentiate into osteoblasts through cellular plasticity to support cortical bone regeneration. **(B)** Central bone marrow of control (*Cxcl12^GFP/+^*) and Col2a1-CXCL12 cKO (*Col2a1-cre; Cxcl12^GFP/fl^*) mice stained for CD45 and lipid TOX, at 3W (left) and 3M (right).

Therefore, Adipo-CAR cells, which are broadly distributed throughout the central marrow space, may maintain the potential to dedifferentiate into skeletal stem cell-like cells under regenerative conditions, supporting the theory that dormant adipocyte-like marrow stromal cells can support the remarkable regenerative capacity of bones through cellular plasticity. These findings also suggest that skeletal stem cells that are actively involved in regenerative conditions may be at least in part contributed through cellular plasticity in adults (**Figure 2A**) (10, 52).

Additionally, another preadipocyte-like subset of BMSCs termed marrow adipogenic lineage precursor (MALP) cells, which are marked by “adipocyte-specific” lines such as *Adipoq-cre* and *Adipoq-creER*, regulates bone formation within marrow space (12). MALP cells form a vast three-dimensional network surrounding sinusoidal blood vessels, and ablation of these cells using a diphtheria toxin fragment A (DTA) allele causes a massive increase of trabecular bones throughout marrow space, especially in female mice (12, 53). This is considered to arise from loss of MALP cell-derived factors that locally inhibit differentiation of skeletal stem/progenitor cells. Therefore, MALP cells can regulate osteogenesis in a cell non-autonomous manner.

Therefore, multiple adipocyte-related cell types have been described to date in the bone marrow, and these adipogenic subsets of BMSCs possess unexpectedly diverse functions beyond formation of marrow adipose tissues, particularly in the context of cortical and trabecular bone formation and regeneration. The important remaining question is whether these thus-far identified adipogenic BMSC subsets represent separate or overlapping entities, or reside in distinct bone marrow microenvironments in a way consistent with two different classes of BMATs. Delineating adipogenic BMSC subsets further will facilitate our understanding of diverse functions of bone marrow adipocytes and their precursor cells.

## CXCL12-Mediated Cell Non-Autonomous Mechanisms Direct Bone Marrow Adipogenesis

The prevailing notion is that bone marrow adiposity is induced by an aberrant cell fate shift of bone marrow skeletal stem cell populations due to cell-intrinsic changes. For example, loss of Wnt/β-catenin signaling (54), intracrine VEGF signaling (55) or PTH/PTHrP receptor signaling (56) and its downstream Gsα signaling (57), has been shown to induce a bias in cell differentiation toward adipocytes. However, because there are a large number of preadipocyte-like cells within the stromal compartment throughout the marrow space and these cells have not yet accumulated intracellular lipid, marrow adiposity may be more promptly regulated at the transition between these precursor cells to fully lipid-laden marrow adipocytes. In other words, the rate-limiting step for marrow adiposity may be at the level of precursor cells, but not at the level of stem cells.

CXCL12 has well-documented roles in hematopoiesis. Additionally, CXCL12 released by BMSCs regulates osteogenesis and adipogenesis; while osteogenesis is controlled by a cell-autonomous mechanism mediated by the CXCL12-CXCR4 signaling pathway, adipogenesis is regulated by a cell-non-autonomous mechanism involving an unidentified cell type (50). The Lai group demonstrates that conditional deletion of *Cxcl12* using *Prrx1-cre* or *Osx-cre* leads to marrow adiposity and reduced trabecular bones, whereas conditional deletion of *Cxcr4* using *Prrx1-cre* causes reduced trabecular bones without marrow adiposity. Interestingly, marrow adiposity in these *Cxcl12*-deficient mice occurs in the metaphyseal region starting from neonatal stages (50). These premature bone marrow adipose tissues in the mutant mice resemble rBMAT, indicating that rBMAT formation may be at least in part regulated by non-cell autonomous mechanisms.

The next important question is, what are the potential cell non-autonomous mechanisms that regulate premature marrow adiposity associated with CXCL12-deficient BMSCs? More recent studies demonstrate that many CXCL12^+^ preadipocyte-like cells (Cxcl12-creER^+^, Adipo-CAR cells) are physically coupled with hematopoietic cells, particularly with B-cell precursors, monocytes and granulocytes, in a protease-sensitive manner, possibly through CXCL12-CXCR4 interactions (51). Further, CXCL12 deletion in BMSCs using *Col2a1-cre* leads to a reduction of stromal-hematopoietic coupling and extensive marrow adipogenesis specifically in adult bone marrow (**Figure 2B**). Interestingly, unlike other CXCL12-deficient models, marrow adiposity does not occur in younger stages in these *Col2a1-cre*-driven *Cxcl12* mutant mice, with Adipo-CAR cells appearing to directly convert into lipid-laden adipocytes without involving cell-intrinsic defects in skeletal stem/progenitor cell fates.

Therefore, these studies indicate that there may be a potential link between CXCL12-dependent stromal-hematopoietic coupling and marrow adipogenesis, as the adipogenic subset of CXCL12^+^ BMSCs are highly interactive with hematopoietic cells. One possible mechanism is that physically-coupled hematopoietic cells provide some types of microenvironmental cues to prevent premature differentiation of pre-adipocyte-like stromal cells into fully lipid-laden marrow adipocytes (50, 51). It is intriguing to think that hematopoietic cells may directly or indirectly regulate marrow adipogenesis in a manner dependent on CXCL12-mediated physical coupling, although details need to be clarified by further experimentation (**Figure 2**). This may serve as one of the important mechanisms that dictate age and disease-related increase in marrow adiposity commonly observed in humans.

## Bone-Specific Secondary Adipogenesis Pathway: From Osteo-CAR to Marrow Adipocytes?

Interestingly, recent studies demonstrate that there might be an alternative bone-specific adipogenic pathway that bypasses classical adiponectin-expressing preadipocyte-like cells. This unique adipogenic pathway has been discovered in fat-free (FF) mice, in which essentially all adiponectin-expressing adipocytes and their precursor cells are ablated using *Adipoq-cre* and inducible DTA expression (58). This genetic model mimics Berardinelli-Seip congenital generalized lipodystrophy, which is associated with increased bone mass and diabetic conditions. Interestingly, in these lipodystrophic FF mice, bone marrow adipocytes still develop within regions of bone marrow that are normally devoted to hematopoiesis under aging and states of metabolic stresses. These bone marrow adipocytes are recruited from adiponectin-negative stromal cells and specialized for lipid storage with compromised lipid mobilization and cytokine expression.

What is the identity of adiponectin-negative stromal cells that can give rise to special bone marrow adipocytes under lipodystrophic conditions? One possibility is that bone marrow adipocytes can originate alternatively from alternative preosteoblast-like subsets of BMSCs, such as Osteo-CAR cells that localize to the peripheral arterioles and endocortical surfaces, or bone-lining cells that are marked by a pulse-chase protocol using *Dmp1-creER* (59) (**Figure 2A**), although neither of these studies (53, 59) provided direct evidence based on rigorous *in vivo* lineage-tracing approaches that can directly target these cells. These potentially diverse cellular sources of bone marrow adipocytes highlight the adaptability of BMATs, particularly “regulated” rBMAT in bone marrow, which can promptly respond to systemic and local health and disease conditions (17). In fact, these ectopic lipodystrophic marrow adipocytes develop in the regions that normally develop rBMATs, indicating an association between adiponectin-negative adipocyte precursor cells and rBMAT formation. It remains to be determined if preosteoblast-like subsets of BMSCs (such as Osteo-CAR cells) can be directly converted to bone marrow adipocytes in rBMAT under physiological conditions.

## Limitations of Current Studies and Future Directions

It is evident that bone marrow adipocytes constituting the two types of BMATs originate from BMSC subsets. Therefore, the unique metabolic and functional features of bone marrow adipocytes are likely to be conferred by their precursor cell populations. Single-cell RNA-seq studies reveal that preadipocyte-like BMSCs do not represent a single homogenous entity, but are instead constituted by a spectrum of heterogeneous cell types spanning over preosteoblasts and lipid-laden adipocytes. The reasonable hypothesis is that the two distinct classes of BMATs are formed by different types of bone marrow adipocyte precursor cells. However, detailed characteristics of these adipocyte precursor cell subtypes have not been successfully revealed.

Despite tremendous progress in the field in recent years, in-depth functional analyses of bone marrow adipocytes have been hampered due to critical technical limitations. First, bone marrow adipocytes are extremely fragile and large-sized, therefore there is no reliable method to isolate these cells for single-cell analyses or culture them *in vitro*. Second, no transgenic tool is available yet to target only adipocytes and their precursors in bone marrow, but not those in subcutaneous tissues or elsewhere. Because of these technical hurdles, the functional significance of bone marrow adipocytes in bone metabolism and hematopoiesis has not been fully uncovered. Third, an inducible genetic tool that can specifically mark preosteoblast-like subsets of BMSCs, such as Osteo-CAR cells, is not yet available. Therefore, whether an alternative bone-specific adipogenesis pathway truly exists has not been formally demonstrated.

Technical breakthroughs are needed to unravel intercellular interactions among bone marrow adipocytes and their surrounding cells at a single-cell level in their native environment. Bone marrow adipocytes constitute a part of the stromal-reticular network, residing in an intricate microenvironment (60) and abundantly secreting a large variety of cytokines including SCF and CXCL12. More sophisticated approaches will be needed to fully define the functions of different types of bone marrow adipocytes and their precursor cells *in vivo*, and eventually define how they regulate bone metabolism and hematopoiesis through both cell-autonomous and cell-non-autonomous mechanisms.

## Conclusion

In this review, we discussed the current understanding of the bone marrow stromal cell lineage, which is largely contributed by recent scRNA-seq and *in vivo* lineage-tracing studies. These studies have substantially refined our idea on bone marrow adipocytes and their precursor cells, particularly highlighting important functions of the adipogenic subsets of bone marrow stromal cells in marrow adiposity and bone homeostasis.

It has been generally considered that skeletal stem cells have pivotal roles as a cellular origin of bone marrow adipocytes. While this concept still holds true, the emerging notion is that the important regulatory step may lie downstream at the level of adipocyte precursor cells; this is facilitated by the discovery of a discrete preadipocyte-like population of CXCL12^+^LepR^+^ BMSCs that exist in a large number throughout the marrow space. Functionally important subsets of adipogenic BMSCs include recently described populations of Adipo-CAR cells and MALPs that have substantial regulatory functions in osteogenesis. Prompt increase in marrow adiposity in aging and other disease conditions may be induced by virtue of the inherent capacity of adipogenic BMSCs that can easily convert to lipid-laden bone marrow adipocytes.

The trajectory to bone marrow adipocytes is now becoming clearer. The remaining task is to clarify if there are discrete precursor cell populations that contribute to two types of BMATs. It is possible that bone marrow adipocytes of “regulated” rBMAT are also contributed to by an alternative bone-specific pathway originating from preosteoblasts, although this pathway has been demonstrated so far only in extreme adipocyte scarcity in lipodystrophic fat-free mice. Clarifying further cellular origins of bone marrow adipocytes of cBMAT and rBMAT will give us the opportunity to clarify the formation and the disappearance of marrow adipocytes in normal development, pathological conditions and therapeutic responses. These future endeavors will lead to a more detailed understanding of the function of bone marrow adipocytes that have incredibly diverse functions through local and systemic regulations.

## Author Contributions

YM and NO drafted the manuscript and generated the figure. YM conducted literature search. WO critically revised the manuscript. All approved the final version of the manuscript.

## Funding

This research was supported by National Institute of Health grants R01DE026666 and R01DE030630 (to NO) and R01DE029181 (to WO)

## Conflict of Interest

The authors declare that the research was conducted in the absence of any commercial or financial relationships that could be construed as a potential conflict of interest.

## Publisher’s Note

All claims expressed in this article are solely those of the authors and do not necessarily represent those of their affiliated organizations, or those of the publisher, the editors and the reviewers. Any product that may be evaluated in this article, or claim that may be made by its manufacturer, is not guaranteed or endorsed by the publisher.
